# The identification and functional implications of human-specific "fixed" amino acid substitutions in the glutamate receptor family

**DOI:** 10.1186/1471-2148-9-224

**Published:** 2009-09-08

**Authors:** Hiroki Goto, Kazunori Watanabe, Naozumi Araragi, Rui Kageyama, Kunika Tanaka, Yoko Kuroki, Atsushi Toyoda, Masahira Hattori, Yoshiyuki Sakaki, Asao Fujiyama, Yasuyuki Fukumaki, Hiroki Shibata

**Affiliations:** 1 Division of Human Moelcular Genetics, Research Center for Genetic Information, Medical Institute of Bioregulation, Kyushu University, Fukuoka 812-8582, Japan; 2 RIKEN Genomic Sciences Center, 1-7-22 Suehiro-cho, Tsurumi-ku, Yokohama, Kanagawa 230-0045, Japan; 3 RIKEN Advanced Science Institute (ASI), Advanced Computational Sciences Department, Computational Systems Biology Research Group, Synthetic Biology Team, Suehiro-cho 1-7-22, Tsurumi-ku, Yokohama, Kanagawa, Japan; 4 Comparative Genomics Laboratory, National Institute of Genetics, Yata 1111, Mishima, Shizuoka 411-8540, Japan; 5 Graduate School of Frontier Sciences, University of Tokyo, 5-1-5 Kashiwanoha, Kashiwa, Chiba 277-8561, Japan; 6 National Institute of Informatics, 2-1-2 Hitotsubashi, Chiyoda-ku, Tokyo, 101-8430, Japan

## Abstract

**Background:**

The glutamate receptors (GluRs) play a vital role in the mediation of excitatory synaptic transmission in the central nervous system. To clarify the evolutionary dynamics and mechanisms of the GluR genes in the lineage leading to humans, we determined the complete sequences of the coding regions and splice sites of 26 chimpanzee GluR genes.

**Results:**

We found that all of the reading frames and splice sites of these genes reported in humans were completely conserved in chimpanzees, suggesting that there were no gross structural changes in humans after their divergence from the human-chimpanzee common ancestor. We observed low *K*_*A*_/*K*_*S *_ratios in both humans and chimpanzees, and we found no evidence of accelerated evolution. We identified 30 human-specific "fixed" amino acid substitutions in the GluR genes by analyzing 80 human samples of seven different populations worldwide. Grantham's distance analysis showed that *GRIN2C *and *GRIN3A *are the most and the second most diverged GluR genes between humans and chimpanzees. However, most of the substitutions are non-radical and are not clustered in any particular region. Protein motif analysis assigned 11 out of these 30 substitutions to functional regions. Two out of these 11 substitutions, D71G in *GRIN3A *and R727H in *GRIN3B*, caused differences in the functional assignments of these genes between humans and other apes.

**Conclusion:**

We conclude that the GluR genes did not undergo drastic changes such as accelerated evolution in the human lineage after the divergence of chimpanzees. However, there remains a possibility that two human-specific "fixed" amino acid substitutions, D71G in *GRIN3A *and R727H in *GRIN3B*, are related to human-specific brain function.

## Background

Glutamate is the most abundant fast-excitatory neurotransmitter in the central nervous system (CNS) and glutamate receptors (GluRs) play a vital role in the mediation of excitatory synaptic transmission. Because of their roles in neurotransmission and synaptic plasticity, GluRs are thought to be key molecules in cognitive functions such as learning and memory (reviewed in [1-3]). Based on their structural and functional characteristics, GluRs are classified into two major groups: ionotropic glutamate receptors (iGluRs) and metabotropic glutamate receptors (mGluRs) (Reviewed in [3]). Vertebrate iGluRs are pharmacologically classified into four subgroups by their ligand selectivity: NMDA, AMPA, kainate, and delta. While iGluRs directly regulate the ion flux across the cell membrane as ion channels, mGluRs are involved in a variety of intracellular signaling pathways by activating phospholipase C and/or suppressing adenlylate cyclase and subsequently mediating excitatory neurotransmission and synaptic plasticity by affecting iGluR activities.

Recent studies have reported that the genetic variations in GluRs are associated with multiple neurobehavioral phenotypes in humans including addictions, anxiety/dysphoria disorders, schizophrenia, and epilepsy [*e.g*., [4-12]]. These observations suggest that genetic variations in GluRs cause brain dysfunctions in humans. A study of the evolutionary genetic changes in the GluR genes would provide us with insights into the molecular basis of human-specific brain and nervous system functions.

The evolutionary changes that occurred in the GluR genes in the lineage leading to humans are poorly understood, mainly because of the limited availability of relevant information in public databases. To overcome this problem, we determined the complete coding sequences of 26 GluR genes in chimpanzees (*Pan troglodytes*) and conducted a comparative genomic analysis of all GluR genes. We examined whether positive selection plays a role in the evolution of the GluR gene family after the divergence of humans and chimpanzees and investigated human-specific "fixed" nonsynonymous substitutions in the GluR genes that might be associated with human-specific brain function.

## Results

We determined the complete nucleotide sequences of the coding exons for 21 chimpanzee glutamate receptor (GluR) genes: *GRIA3, GRIA4, GRID1, GRID2, GRIK1, GRIK2, GRIK3, GRIK4, GRIK5, GRIN1, GRIN2A, GRIN2C, GRIN2D, GRIN3A, GRIN3B, GRM1, GRM2, GRM4, GRM6, GRM7*, and *GRM8*. The sequences of five additional chimpanzee GluR genes, *GRIA1, GRIA2, GRIN2B, GRM3*, and *GRM5 *were obtained from the UCSC Genome Database (version panTro1) [13]. Successful alignments of the entire coding regions and splice sites of the human and chimpanzee GluR genes indicated no gross structural differences such as protein truncation between humans and chimpanzees.

### The pairwise nucleotide divergence

We calculated the pairwise nucleotide divergence in total and synonymous sites between humans and chimpanzees for all of the genes encoding NMDA, AMPA, kainate, delta, and metabotropic glutamate receptors (Table 1). The divergence for the entire set of GluR genes both at total and synonymous sites was 0.00461 and 0.01257, respectively, which is significantly lower than the genome-wide average values, which are 0.0059 and 0.0177 (more than 2 SD below the mean in a normal distribution, [14]). Indeed, except for the NMDA type, the divergence for each type was significantly lower than the genome-wide average at total and synonymous sites (more than 2 SD below the mean).

**Table 1:** The pairwise nucleotide divergence per site at total and synonymous sites between humans and chimpanzees

**Type**	**Total sites**	**Synonymous sites**
AMPA	0.00242 ± 0.00053	0.00792 ± 0.00167
Delta	0.00432 ± 0.00076	0.01341 ± 0.00292
Kainate	0.00429 ± 0.00047	0.01288 ± 0.00166
NMDA	0.00594 ± 0.00045	0.01477 ± 0.00141
mGluR	0.00444 ± 0.00041	0.01186 ± 0.00122

All GluRs	0.00461 ± 0.00019	0.01257 ± 0.00056

The AMPA type genes showed the lowest divergence of the GluR types at both total and synonymous sites (0.00242 ± 0.00053 and 0.00792 ± 0.00167 at total and synonymous sites, respectively). The divergence of the four individual AMPA genes ranged from 0.00075 to 0.00303 at total sites and from 0.00056 to 0.00103 at synonymous sites. Therefore, the AMPA type genes showed lower divergence than the other GluR types. The divergence for the NMDA type genes was the highest of all of the GluR types at both total and synonymous sites (0.00594 ± 0.00045 and 0.01477 ± 0.00141). This observed higher divergence can be attributed to two NMDA type GluR genes, *GRIN3A *and *GRIN3B*. The *GRIN3B *and *GRIN3A *genes are the most and the second most diverged GluR genes between humans and chimpanzees at both types of site (0.01351 and 0.00965 at total sites and 0.03023 and 0.02844 at synonymous sites). When we excluded *GRIN3A *and *GRIN3B *from this analysis, the NMDA type genes were found to have similar divergences to the other gene types at total and synonymous sites (0.00425 ± 0.00040 and 0.01154 ± 0.00135).

### Lineage-specific K_A_/K_S _ratios and selection tests

Intrigued by the possible functional implications of the GluR genes in the evolutionary process of the human lineage, we examined whether positive selection acted on the GluR genes in humans and chimpanzees. At first, using macaque sequences as an outgroup, we calculated the human and chimpanzee lineage-specific nonsynonymous and synonymous substitution rates (*K*_*A *_and *K*_*S*_, respectively) and their ratios (*K*_*A*_/*K*_*S*_, Table 2). Notably, the *K*_*A*_/*K*_*S *_ratios for the GluR genes were less than one in both the human and chimpanzee lineages, although we could not calculate the ratios for 11 human and 11 chimpanzee GluR genes due to the absence of nonsynonymous substitutions. Excluding the genes with no substitutions, the *K*_*A*_/*K*_*S *_ratios ranged from 0.0004 to 0.417 and 0.0005 to 0.242 in human and chimpanzee lineages, respectively. The *K*_*A*_/*K*_*S *_ratios for 24 human GluR genes and all of the chimpanzee GluR genes are smaller than the species-specific genome-wide mean values (0.259 and 0.245 in human and chimpanzee, respectively [15]). These results indicate that the functional constraint on the GluR is relatively strong.

**Table 2:** The nonsynonymous and synonymous rates and their ratio in the human and chimpanzee lineages

	**Human**			**Chimpanzee**		
		
**gene**	***K*_*A*_**	***K*_*S*_**	***K*_*A*_/*K*_*S*_**	***K*_*A*_**	***K*_*S*_**	***K*_*A*_/*K*_*S*_**
*GRIA1*	0	0.0051	0	0	0.0053	0
*GRIA2*	0	0.0041	0	0	0.0069	0
*GRIA3*	0	0.0012	0	0.0005	0	0
*GRIA4*	0.0006	0.0059	0.0933	0	0.0023	0
*GRID1*	0.0009	0.0093	0.0947	0	0.007	0
*GRID2*	0	0.0109	0	0.0005	0.0032	0.1495
*GRIK1*	0	0.0024	0	0	0.0106	0
*GRIK2*	0	0.0028	0	0	0.0043	0
*GRIK3*	0.0005	0.0189	0.0247	0.0005	0.0197	0.0236
*GRIK4*	0.0004	0.0139	0.0323	0	0.0064	0
*GRIK5*	0.0013	0.0055	0.2313	0.0004	0.0041	0.1025
*GRIN1*	0	0.0159	0	0	0.0051	0
*GRIN2A*	0.0009	0.0066	0.1396	0.0009	0.0075	0.1244
*GRIN2B*	0	0.0078	0	0.0003	0.0058	0.0518
*GRIN2C*	0.0023	0.013	0.1745	0.0015	0.0129	0.1157
*GRIN2D*	0	0.013	0	0	0.005	0
*GRIN3A*	0.0026	0.0087	0.2976	0.0017	0.015	0.1131
*GRIN3B*	0.0017	0.0447	0.0388	0	0	0
*GRM1*	0.0004	0.0082	0.0466	0.0004	0.0042	0.0901
*GRM2*	0.0005	0.0108	0.049	0.0005	0.0074	0.0709
*GRM3*	0.0005	0.0013	0.417	0.0011	0.0044	0.2422
*GRM4*	0	0.0109	0	0.0005	0.0069	0.0663
*GRM5*	0	0.0031	0	0	0.0057	0
*GRM6*	0.0025	0.0224	0.1123	0.0016	0.0159	0.1038
*GRM7*	0.0005	0.0105	0.0514	0.0011	0.0071	0.1521
*GRM8*	0.0005	0.0039	0.1327	0.001	0.0055	0.1902

*K*_*A*_, *K*_*S*_, and *K*_*A*_/*K*_*S *_indicate nonsynonymous, synonymous, and the nonsynonymous/synonymous substitution rate (per site), respectively.

Two maximum likelihood ratio tests (implemented in PAML [16]) were employed to examine the *K*_*A*_/*K*_*S *_for the GluR genes in the human lineage and to evaluate the accelerated selection for the GluR genes in humans and chimpanzees. First, we compared the *K*_*A*_/*K*_*S *_ratios in the human lineage *vs*. background (chimpanzee and macaque) lineages. We observed that the human-specific *K*_*A*_/*K*_*S *_ratio is significantly different from the background ratio in *GRM7 *according to test B outlined in [17] (Additional file 1). Taking the *K*_*A*_/*K*_*S *_value into account, this result implies that the human *K*_*A*_/*K*_*S *_ratio is significantly lower than that of the background in *GRM7*. We then applied the improved branch-site model [18] to examine whether positive selection acted on the GluR genes in humans and chimpanzees, but could not detect any significant accelerated selection in either human or chimpanzee lineages (Additional file 2).

### Identification of human-specific "fixed" nonsynonymous mutations

To evaluate the functional changes of the GluR genes in the human lineage, we searched for human-specific "fixed" nonsynonymous substitutions. First, we carried out a pairwise comparison between the human and chimpanzee orthologs of 26 GluR genes. We found a total of 80 nonsynonymous substitutions including four indels (insertion/deletion) (Table 3). Out of the 80 substitutions, two substitutions were excluded from further analysis due to discrepancies among the UCSC reference and our chimpanzee sequences; these two substitutions are possibly polymorphic within chimpanzees. Second, we sequenced the remaining 78 substitution/indel sites in five additional apes: the bonobo, gorilla, orangutan, siamang, and crab-eating macaque. We regarded human alleles that were not shared with any of the great apes to be "human-specific". We pooled substitutions that were found specifically either in chimpanzees or in bonobos as "chimpanzee-specific" substitutions, because these mutations must have occurred in the chimpanzee and bonobo lineages after the divergence of the human lineage. Out of the 78 human-chimpanzee substitution sites, we identified 37 human-specific (35 substitutions and 2 indels) and 31 chimpanzee-specific substitutions/indels (29 substitutions and 2 indels). The remaining 10 are recurrent substitutions in the primate lineages. To determine whether these substitutions/indels are "fixed" or "polymorphic" in human populations, we sequenced 80 human samples representing seven different populations around the world for the 37 human-specific substitution/indel sites. Table 3 shows the 30 "fixed" (28 substitutions and 2 indels) and 7 "polymorphic" substitutions/indels that we confirmed in the human populations. The 30 human-specific "fixed" substitutions are potentially responsible for human-specific functions.

**Table 3:** Comparison of human-chimpanzee substitutions among primates

**ID#**	**Gene**	**Amino acid (Hum-Chimp)**	**Nucleotide (Hum-Chimp)**	**Hum**	**Chimp**	**Bon**	**Gor**	**Ora**	**Gib**	**Mac #1**	**Mac #2**	**Specificity**^1^	**Fixed in Humans**	**Notes**
1	*GRIN2A*	S906N	AGC-AAC	**G**	A	A	A	A	A	A	A	Human	Fixed	
2	*GRIN2A*	A1006V	GCG-GTG	**C**	T	T	T	T	T	T	T	Human	Fixed	
3	*GRIN2A*	H1080P	CAC-CCC	A	**C**	A	A	A	A	A	A	Chimpanzee		
4	*GRIN2A*	F1158L	TTC-TTG	G	**C**	**C**	G	G	G	G	G	Chimpanzee		
5	*GRIN2A*	H1173Q	CAT-CAA	A	**T**	**T**	A	A	A	A	A	Chimpanzee		
6	*GRIN2A*	M1221L	ATG-CTG	**A**	C	C	C	C	C	C	C	Human	Fixed	

7	*GRIN2B*	N1294T	AAC-ACC	A	**C**	**C**	A	A	A	A	A	Chimpanzee		

8	*GRIN2C*	P23L	CCG-CTG	CCG	ATG	CTG	CCG	CTG	CGG	CCG	CCG	N.A.		
9	*GRIN2C*	T71N	ACC-AAC	**C**	A	A	A	**C**	**C**	A	A	N.A.		
10	*GRIN2C*	H89R	CAC-CGC	**A**	G	G	G	G	G	G	G	Human	Fixed	
11	*GRIN2C*	D100G	GAC-GGC	**A**	G	G	G	G	G	G	G	Human	Fixed	
12	*GRIN2C*	A596S	GCT-TCT	**G**	T	T	T	"T"	T	T	T	Human	Fixed	
13	*GRIN2C*	S851T	TCC-ACC	T	**A**	**A**	T	T	T	T	T	Chimpanzee		
14	*GRIN2C*	Q898R	CAG-CGG	A	**A/G**	-	A	A	A	A	A	Chimpanzee		
15	*GRIN2C*	S933P	TCC-CCC	**T**	C	-	-	C	C	"C"	"C"	Human	Fixed	
16	*GRIN2C*	G1144S	GGC-AGC	G	**A**	**A**	G*	G	G	"G"	"G"	Chimpanzee		*AGG
17	*GRIN2C*	R1221C	CGT-TGT	**C**	T	T	-	"T"	T	"T"	"T"	Human	Fixed	

18	*GRIN3A*	S30G	AGC-GGC	**A**	G	G	-	"G"	G	G	G	Human	Fixed	
19	*GRIN3A*	D71G	GAC-GGC	**A**	G	G	-	"G"	G	G	G	Human	Fixed	
20	*GRIN3A*	P93L	CCG-CTG	C	**T**	**T**	C	C	C	C*	C*	Chimpanzee		*TCG
21	*GRIN3A*	A119T	GCG-ACG	G	**A**	G	G	G	G	G	G	Chimpanzee		
22	*GRIN3A*	A121T	GCC-ACC	**G**	A	A	A	A	A	A	A	Human	Fixed	
23	*GRIN3A*	V138M	GTG-ATG	G	**A**	G	G	G*	G*	G*	G	Chimpanzee		*GGG
24	*GRIN3A*	E340K	GAA-AAA	G	**A**	G	G	G	G	G	G	Chimpanzee		
25	*GRIN3A*	A885S	GCC-TCC	**G**	T	T	T	T	T	T	T	Human	Fixed	
26	*GRIN3A*	I988V	ATA-GTA	**A**	G	G	G	G	G	G	G	Human	Fixed	
27	*GRIN3A*	R1059L	CGG-CTG	**G**	T	T	-	T	T	T	T	Human	Fixed	

28	*GRIN3B*	P17S	CCG-TCG	C	**T**	**T**	C	C	C	C	C	Chimpanzee		
29	*GRIN3B*	G175S	GGC-AGC	G	**A**	G	-	G	G	G	G	Chimpanzee		
30	*GRIN3B*	E229G	GAA-GGA	A	**G**	A	-	A	A	-	A	Chimpanzee		
31	*GRIN3B*	A272V	GCG-GTG	C	**T**	C	C	C*	C	C*	C*	Chimpanzee		*GCA
32	*GRIN3B*	I296T	ATT-ACT	**T**	C	C	C*	C	C*	C/T**	C*	N.A.		*ACG, **ATT/CCT
33	*GRIN3B*	W414R	TGG-CGG	**T**	C	C	C	C	C	C	C	Human	NOT	rs2240157
34	*GRIN3B*	A468V	GCG-GTG	**C**	T	T	T	T	T	T	T	Human	Fixed	
35	*GRIN3B*	R473C	CGC-TGC	C	**T**	**T**	C	C	C	C	C	Chimpanzee		
36	*GRIN3B*	L499I	CTC-ATC	**C**	A	A	A	A	A	A	A	Human	Fixed	
37	*GRIN3B*	T577M	ACG-ATG	**C**	T	T	T	T	-	T	T	Human	NOT	rs2240158
38	*GRIN3B*	Y595C	TAC-TGC	A	**G**	**G**	**G**	A	-	A	A	N.A.		
39	*GRIN3B*	R598C	CGT-TGC	CGT	**TGC**	**TGC**	**TGC**	CGC	-	CGC	CGC	N.A.		
40	*GRIN3B*	V613I	GTC-ATC	G	**A**	G	G	G	-	G	G	Chimpanzee		
41	*GRIN3B*	R727H	CGC-CAC	**G**	A	A	A	A	A	"A"	"A"	Human	Fixed	

42	*GRIA3*	P590L	CCT-CTT	C	**T**	C	C	C	C	C	C	Chimpanzee		

43	*GRIA4*	S5C	TCC-TGC	**C**	G	G	G	G	G	G	G	Human	Fixed	

44	*GRIK3*	S310A	TCC-GCC	**T**	G	G	G	"G"	G	G	G	Human	NOT	rs6691840
45	*GRIK3*	V419I	GTT-ATT	G	**A**	**A**	G	G	G	G	G	Chimpanzee		

46	*GRIK4*	H403R	CAC-CGC	**A**	G	G	G	**A**	G	G	G	N.A.		

47	*GRIK5*	L298P	CTG-CCG	**T**	C	C	C	C	C	C	C	Human	Fixed	
48	*GRIK5*	I809V	ATC-GTC	**A**	G	G	G	"G"	-	G	G	Human	Fixed	*GTT
49	*GRIK5*	A922T	GCC-ACC	G	**A**	-	-	G	G	G	G	Chimpanzee		
50	*GRIK5*	V956A	GTC-GCC	**T**	C	**C/T**	-	**C/T**	-	C	C	N.A.		

51	*GRID1*	T295M	ACG-ATG	**C**	T	T	T	T	-	T	T	Human	Fixed	
52	*GRID1*	M628V	ATG-GTG	**A**	G	G	G	G	G	G	G	Human	Fixed	

53	*GRID2*	S11F	TCC-TTC	C	**T**	**T**	C	C	C	C	C	Chimpanzee		

54	*GRM1*	S993P	TCC-CCC	**T**	C	C	C	C	C	C	C	Human	NOT	rs6923492
55	*GRM1*	L1089P	CTG-CCG	**T**	C	C	C	C	C	**T**	**T**	N.A.		*CCA

56	*GRM2*	A6G	GCG-GGG	**G**	C	C	C	C	C	C	C	Human	Fixed	
57	*GRM2*	A248V	GCG-GTG	C	**T**	**T**	C	C	C	C	C	Chimpanzee		

58	*GRM3*	M547V	ATG-GTG	A	**G**	**G**	A	A	A	A	A	Chimpanzee		
59	*GRM3*	S551P	TCT-CCT	**T**	C	C	C	C	C	C	C	Human	NOT	No rs# available
60	*GRM3*	M593T	ATG-ACG	T	**C/T**	T	T	T	T	T	T	Chimpanzee		

61	*GRM4*	L19F	CTC-TTC	C	**T**	**T**	C	C	C	C	C	Chimpanzee		

62	*GRM6*	Q59P	CAG-CCG	**A**	C	C	C	C	C	C	C	Human	NOT	rs2645329
63	*GRM6*	P141T	CCC-ACC	**C**	A*	A	A	A	A	A	-	Human	NOT	No rs # available *AT/CC
64	*GRM6*	D380E	GAT-GAG	T	**G**	**G**	T	T	T	T	T	Chimpanzee		
65	*GRM6*	M442T	ATG-ACG	**T**	C	C	C	C	**T**	C	C	N.A.		
66	*GRM6*	Y612H	TAC-CAC	**T**	C	C	C	C	C	C	C	Human	Fixed	
67	*GRM6*	A650G	GCG-GGG	C	G	G	C	C	C	T	T	N.A.		
68	*GRM6*	M714V	ATG-GTG	**A**	G	G	G*	G	G	G	G	Human	Fixed	*GCG
69	*GRM6*	V839I	GTA-ATA	G	**A**	**A**	G	G	G	G	G	Chimpanzee		
70	*GRM6*	A877D	GCC-GAC	**C**	A	A	A	A	A	A	A	Human	Fixed	

71	*GRM7*	A520P	GCC-CCC	**C**	G	G	G	G	G	G	G	Human	Fixed	

72	*GRM8*	R268C	CGC-TGC	A	**G**	A	A	A	A	A	A	Chimpanzee		
73	*GRM8*	G327V	GGG-GTG	G	**G/T**	**G/T**	G	G	G	G	G	Chimpanzee		
74	*GRM8*	V653I	GTC-ATC	**G**	A	A	A	A	A	A	A	Human	Fixed	

75	*GRIN2C*	del1021-1026RALPER	CGCGCGCTCCCAGAGCGG	**del**	in	in	-	in	in	in	in	Human	Fixed	
76	*GRIN2C*	PPE 1055-1057del	CCCCCGGAG	in	**in/del**	in	-	"in"	in	in	in	Chimpanzee		
77	*GRIN2C*	AH1164-1165del	GCCCAC	in	**in/del**	in	in	in	in	in	-	Chimpanzee		

78	*GRM6*	GD125-126del	GCGACG	**in**	del	del	-	del	del	del	-	Human	Fixed	

Abbreviations are: Hum - human, Chimp - chimpanzee, Bon - bonobo, Gor - gorilla, Ora - orangutan, Gib - gibbon, in - insertion, del -- deletion, rs -- RefSNP accession ID.^1^: This shows the lineage in which the substitution (insertion/deletion) occurred. In cases in which the substitution independently occurred at the same site in more than two lineages, the specificity is not assigned (N.A.). We pooled substitutions found specifically in chimpanzees or in bonobos as "chimpanzee-specific" substitutions.

" ": The sequence was obtained from the UCSC genome database.

-: We failed to determine the sequence of the site, and the sequence was not available from the UCSC genome database.

*: The other substitution was found in the codon.

### Functional implications of human-specific "fixed" nonsynonymous substitutions

To evaluate the functional significance of each amino acid substitution in the GluR genes, we calculated Grantham's distance [19], a measurement of the chemical drasticity of amino acid replacements. We examined the differences in amino acid substitution patterns between humans and chimpanzees using 35 human-specific and 29 chimpanzee-specific substitutions after excluding 4 indel sites. We classified amino acid substitutions into two groups: substitutions with Grantham's distances greater than 100 (the mean chemical distance from the three-property formula [19]) were classified as "radical" changes and substitutions with Grantham's distance less than 100 were classified as "non-radical" changes. We found four radical and 30 non-radical changes in the human lineage and four radical and 25 non-radical changes in the chimpanzee lineage, indicating that there are no significant differences in the amino acid substitution patterns between humans and chimpanzees (*p *= 1 in Fisher's exact test, 2 × 2, two-tailed). We then summarized Grantham's distance for each GluR gene that contained one of the 28 human-specific "fixed" substitutions (Table 4). Among the 11 GluR genes that have human-specific "fixed" substitutions, Grantham's distance analysis showed that *GRIN2C *and *GRIN3A *are the most diverged in humans from the human-chimpanzee ancestor sequence (476 in *GRIN2C *and 438 in *GRIN3A*). Four out of the five "fixed" substitutions in *GRIN2C *and five out of the six "fixed" substitutions in *GRIN3A *are non-radical changes with Grantham's distances of less than 100. These substitutions are not located in any particular region, but instead are distributed throughout the entire gene regions (Table 4). These results imply that it is unlikely that the accumulation of non-radical substitutions in *GRIN2C *and *GRIN3A *caused their functional divergence from their ancestors.

**Table 4:** Grantham's distance for GluR genes using human-specific "fixed" amino acid substitutions

**Gene**	**Number of substitutions**	**Total Grantham's distance**	**Substitutions** **(Amino acid position, Grantham's distance)**
*GRIN2C*	5	476	(89, 29) (100, 94) (596, 99) (933, 74) (1221, 180)
*GRIN3A*	6	438	(30, 56) (71, 94) (121, 58) (885, 99) (988, 29) (1059, 102)
*GRM6*	3	230	(612, 83) (714, 21) (877, 126)
*GRIK5*	2	127	(298, 98) (809, 29)
*GRIN2A*	3	125	(906, 46) (1006, 64) (1221, 15)
*GRIA4*	1	112	(5, 112)
*GRID1*	2	102	(295, 81) (628, 21)
*GRIN3B*	3	98	(468, 64) (499, 5) (727, 29)
*GRM2*	1	60	(6, 60)
*GRM8*	1	29	(653, 29)
*GRM7*	1	27	(520, 27)

Total	28	1824	

Using the MEMSAT3 [20] and MyHits [21], we annotated the transmembrane and protein motif domains around the 30 "fixed" human-specific substitution/indel sites (Table 5). We found that ten substitutions are located within either transmembrane or protein motif domains: four in transmembrane domain sites, two in N-glycosylation sites, two in N-myristoylation sites, and two in phosphorylation sites. Out of these ten substitutions, there are two human-specific and "fixed" substitutions, D71H in *GRIN3A *and R27H in *GRIN3B*, which alter the functional assignments of their respective genes as determined by the aforementioned annotation software. D71G in *GRIN3A *abolishes an N-myristoylation site that is conserved in other apes and R727H in *GRIN3B *generates a novel phosphorylation site for protein kinase C in the human lineage. These substitutions may cause functional changes in human GluRs that contribute to human brain function.

**Table 5:** Human-chimpanzee amino acid substitutions in functional domains

**Gene**	**Amino acid** **(Human-Chimpanzee)**	**Nucleotide** **(Human-Chimpanzee)**	**Functional Domain**
*GRIN2A*	S906N	AGC-AAC	N-glycosylation site
*GRIN3A*	S30G	AGC-GGC	N-myristoylation site
*GRIN3A*	D71G	GAC-GGC	N-myristoylation site (lost in humans)
*GRIN3A*	I988V	ATA-GTA	Casein kinase II phosphorylation site.
*GRIN3B*	A468V	GCG-GTG	N-glycosylation site
*GRIN3B*	R727H	CGC-CAC	Protein kinase C phosphorylation site (acquired in humans)
*GRIK5*	I809V	ATC-GTC	Transmembrane
*GRM2*	A6G	GCG-GGG	N-myristoylation site
*GRM6*	Y612H	TAC-CAC	Transmembrane
*GRM6*	M714V	ATG-GTG	Transmembrane
*GRM8*	V653I	GTC-ATC	Transmembrane

## Discussion

In this study, we examined the evolutionary changes of the glutamate receptor (GluR) genes in humans and chimpanzees. We found no gross differences in the coding regions or splice sites of the GluR genes between humans and chimpanzees. We also demonstrated that the average rate of protein evolution (*i.e*. the *K*_*A*_/*K*_*S *_ratio) is significantly lower in the GluR genes than the genome-wide average values for humans and chimpanzees. This pattern is consistent with previous genome-wide studies [15,22,23], indicating that strong purifying selection acts on brain-expressed genes including the GluR genes due to their strict functional constraint. There are no significant differences between humans and chimpanzees with regard to their *K*_*A*_/*K*_*S *_values (Table 2) or substitution patterns (Table 4). These results imply that no gross functional changes occurred in either lineage after the human-chimpanzee divergence.

Dorus *et al*. [24] found an increase in the *K*_*A*_/*K*_*S *_ratio of genes involved in the nervous system of primates relative to rodents when housekeeping genes are treated as a control, leading to the conclusion that the primate nervous system genes have experienced accelerated evolution. Our results indicate similar *K*_*A*_/*K*_*S *_ratios between these primate nervous system genes and the GluR genes. Although our *K*_*A*_/*K*_*S *_ratio values for the GluR genes are higher than those of housekeeping genes as discussed in Dorus *et al*. [24], we conclude that the GluR genes have been subject to strong functional constraint rather than rapid positive selection detected as accelerated evolution for the following reasons: First, we could not detect any positive selection for the GluR genes using the statistical tests reported by Dorus *et al*. [24]. Although the statistical power of the tests may have been somewhat affected by the scarcity of substitutions, the improved branch-site test can detect single amino acid substitutions positively selected [18]. Second, there is no local accumulation of amino acid substitutions that could have caused the functional divergence of domains in the GluR genes. Grantham's distance analyses showed that *GRIN2C *and *GRIN3A *are the most and the second most diverged GluR genes between humans and chimpanzees. However, most of the substitutions in these genes are non-radical and are not clustered in any particular region. Third, we identified only two out of the 28 human-specific "fixed" substitutions in the assigned functional transmembrane region. This observation strongly supports severe functional constraint acting on the coding regions of the GluR genes, especially on the functionally important domains.

Niemann *et al*. [25] reported a common null allele of *GRIN3B *with no particular phenotype, indicating relaxed functional constraints on *GRIN3B *in the human lineage. However, we observed a low *K*_*A*_/*K*_*S *_ratio (0.2976) and a low Grantham's distance (98) for human *GRIN3B*. Gene loss or decay might still contribute to functional changes by modifying the genetic network. In fact, NR3B knockout mice have been reported to show highly increased social interaction with their cage mates in their home cage but moderately increased anxiety-like behavior and decreased social interaction in a novel environment [26]. The presence of NR3 in NMDA receptors has been shown to decrease Mg^+2 ^sensitivity and Ca^+2 ^permeability, reduce agonist-induced current responses, and give rise to a new class of excitatory glycine receptors [27]. These observations suggest that NR3 has a significant role in higher brain functions through tetrameric formation with other NR subunits. Two out of the 28 human-specific "fixed" substitutions, D71G in *GRIN3A *and R727H in *GRIN3B*, changed the functional assignments between humans and other apes, causing the loss of a myristoylation site and the gain of a phosphorylation site, respectively. Since myristoylation and phosphorylation are commonly involved in processes related to synaptic plasticity including long-term potentiation and long-term depression in glutamate receptors [28], these two substitutions possibly affect human-specific brain function by modulating NMDA receptor characteristics.

## Conclusion

The results of our comparative genetic study enable us to speculate about the evolutionary changes affecting human-specific brain function that occurred in the GluR genes. We showed that strong purifying selection is the major evolutionary force in the GluR genes shared by humans and chimpanzees. We identified 30 human-specific "fixed" amino acid substitutions/indels including two amino acid substitutions that potentially alter the functional roles of their genes as candidate sites responsible for human-specific brain function. Our results are valuable for understanding the molecular basis of the brain and nervous system in humans and help us to clarify human GluR functions in *in vitro *and *in vivo *experiments.

## Methods

### Sequence data for human, chimpanzee and macaque

Using the longest isoform transcript as a reference (Additional file 3), we retrieved the coding sequences of 26 glutamate receptor (GluR) human and chimpanzee genes from the UCSC Genome Browser (hg18 and panTro2 for humans and chimpanzees, respectively)[12]. Since only partial genomic sequences were available for 21 chimpanzee genes, we determined the chimpanzee sequence for the 21 genes using either PCR-based direct sequencing or a BAC-based cloning-sequencing method (Additional file 3). These genomic GluR sequences were deposited in GenBank (accession numbers: AB514205-AB514225). The genomic sequences of macaque homologs were also obtained from the UCSC Genome Browser (rheMac2).

### DNA samples

Primate DNA samples were kindly provided by Dr. Osamu Takenaka of the Primate Research Institute at Kyoto University and Dr. Takafumi Ishida from the Department of Biological Sciences at the Graduate School of Science of The University of Tokyo. Japanese samples and Thai samples were collected from the Kyushu area of Japan and the Chiang Mai area of Thailand with written informed consent. Other ethnic human samples were purchased from The Coriell Institute for Medical Research [29]. We identified human-specific substitution sites by determining the sequences of five primate species, the bonobo (*Pan paniscus*) gorilla (*Gorilla gorilla *ssp), orangutan (*Pongo pygmaeus pygmaeus*), Siamang (*Symphalangus syndactylus*), crab-eating macaque (*Macaca fascicularis*), and green monkey (*Chlorocebus aethiops*) at the human-chimpanzee substitution sites. Then, we analyzed 80 human DNA samples (Additional file 4), including 7 populations, to confirm "fixation" of these mutations in human species. This study was approved by the Ethics Committee of the Faculty of Medicine at Kyushu University.

### PCR amplification and sequencing

The PCR primers were designed based on the alignments of human and chimpanzee GluR genes using Primer3 [30]. PCR amplification was carried out using 10 μl samples containing 1 μg of genomic DNA, PCR buffer, 2.5 mM dNTPs (Promega, Madison, WI), 0.7 units Taq DNA polymerase (Promega, Madison, WI), 25 mM MgCl_2_, and 10 μM forward and reverse primers. Primer pairs and PCR conditions are described in Additional file 5. After the PCR reaction, we treated the reaction mixtures with 1 U of Exonuclease I (New England Biolabs) and 0.1 U of SAP (Shrimp Alkaline Phosphatase; Roche Applied Science, Indianapolis, IN) to remove the primers.

All sequencing reactions were performed using 10 μl samples containing 1 μl of PCR product, 1.6 μM sequencing primer, and 0.25 μl of BigDye Terminator v1.1 or v.3.1 (Applied Biosystems, Foster City, CA). The conditions for the sequencing reaction were 96°C for 90 seconds, 50°C for 5 seconds, and 60°C for 60 seconds for 25 cycles. The sequencing products were purified by ethanol precipitation and then analyzed on an ABI 3100 or 3730 (Applied Biosystems). Mutation Surveyor v2.2 (SoftGenetics, LLC) was used to compile the electropherograms.

### Data analysis

The pairwise nucleotide divergence was estimated by MEGA [31]. The Tamura and Nei model [32] and Jukes and Cantor model [33] were used for total and synonymous sites, respectively. Standard errors were computed by the bootstrap method (1000 replicates). The nonsynonymous and synonymous lineage-specific rates (*K*_*A *_and *K*_*S*_, respectively) were estimated by the modified Nei and Gojobori [34] and ML [35] methods as implemented in the codeml module of PAML [16].

The Grantham's distances for each substitution were obtained from Table in [19]. Since this distance method is only applicable to amino acid substitutions, we excluded insertion/deletion (indel) sites from this analysis. We used the mean chemical distance from the three-property formula [19], 100, for the cutoff value of two classes, radical and non-radical substitution.

Using PAML software, the likelihood ratio test was applied to examine the following two hypotheses: (1) the *K*_*A*_/*K*_*S *_ratio was significantly higher in the branch of interest than in the background branch (tests B and D in [17]) and (2) positive selection acted on the branch of interest (improved branch site model; test 2 in [18]). The tests were carried out by comparing the log-likelihood values between the null and alternate hypotheses. Bonferroni correction was applied to correct for multiple comparisons.

The topology of transmembrane domains was predicted by the MEMSAT3 module [20] of The PSIPRED Protein Structure Prediction Server [36]. MyHits [21] with the PROSITE database [37] was used to scan all known protein motifs.

## Authors' contributions

HS, HG, and YF designed the study. YF and HS collected human and primate DNA samples. YK, AT, MH, YS, and AF carried out the sequencing of the chimpanzee GluR genes. KW, NA, RK, KT, HS, and HG sequenced the human-chimpanzee substitution sites in humans and the other primates. HG and HS performed data analyses. HG, HS, and YF drafted the manuscript. All authors read and approved the final manuscript.

## Supplementary Material

Additional file 1 **The likelihood statistics used to compare the *K*_*A*_/*K*_*S *_ratios between human and background lineage**. The table shows likelihood ratios and *p *value for the statistical test of the *K*_*A*_/*K*_*S *_ratio comparison between human and background lineage. Click here for file

Additional file 2 **Results for the improved branch site model**. The table presents likelihood ratios and *p *value for the improved branch site test. Click here for file

Additional file 3 **The source of genomic GluR sequences for humans, chimpanzees, and macaques**. The table shows the data source of genomic GluR sequences. Click here for file

Additional file 4 **Human DNA samples used in the polymorphism survey**. The table presents Coriell numbers of human DNA samples for genotyping in human populations. Click here for file

Additional file 5 **Primers and PCR conditions**. The table shows the primer sequences and PCR conditions for genotyping in primates. Click here for file
