# Supplementary material for: The identification and functional implications of human-specific "fixed" amino acid substitutions in the glutamate receptor family
Source: BMC Evol Biol. 2009 Sep 8;9:224. doi: 10.1186/1471-2148-9-224 (PMC2753569; doi:10.1186/1471-2148-9-224)
Supplement: Additional file 1 — The likelihood statistics used to compare the KA/KS ratios between human and background lineage. The table shows likelihood ratios and p value for the statistical test of the KA/KS ratio comparison between human and background lineage. [file 1471-2148-9-224-S1.doc]

**Additional file 1 - The likelihood statistics used to compare the *K_A_*/*K_S_* ratios between human and background lineage**

The background lineage is the chimpanzee-macaque lineage. The left and right sides of the table represent test B and C as described in Table 2 in [17].

|  | Model B | Model A | chi-square value | *p* value (*d.f.*=1) | Model E | Model C | chi-square value | *p* value (*d.f.*=1) |
| --- | --- | --- | --- | --- | --- | --- | --- | --- |
| *GRIA1* | -3904.81 | -3904.81 | 0.000 | 0.998 | -3904.81 | -3904.81 | 0.000 | 0.998 |
| *GRIA2* | -3763.84 | -3763.93 | 0.170 | 0.680 | -3763.93 | -3763.78 | 0.295 | 0.587 |
| *GRIA3* | -3764.92 | -3765.00 | 0.156 | 0.693 | -3765.00 | -3762.14 | 5.707 | 0.017 |
| *GRIA4* | -3794.65 | -3794.97 | 0.643 | 0.423 | -3794.97 | -3794.80 | 0.355 | 0.551 |
| *GRID1* | -4310.26 | -4313.07 | 5.629 | 0.018 | -4313.07 | -4312.89 | 0.357 | 0.550 |
| *GRID2* | -4400.73 | -4401.45 | 1.447 | 0.229 | -4401.45 | -4400.69 | 1.526 | 0.217 |
| *GRIK1* | -4010.69 | -4010.75 | 0.131 | 0.718 | -4010.75 | -4010.44 | 0.616 | 0.433 |
| *GRIK2* | -3456.49 | -3456.57 | 0.157 | 0.692 | -3456.57 | -3456.45 | 0.246 | 0.620 |
| *GRIK3* | -3950.52 | -3950.59 | 0.136 | 0.712 | -3950.59 | -3950.54 | 0.109 | 0.741 |
| *GRIK4* | -3973.20 | -3973.51 | 0.605 | 0.437 | -3973.51 | -3973.29 | 0.438 | 0.508 |
| *GRIK5* | -4103.98 | -4107.06 | 6.154 | 0.013 | -4107.06 | -4106.56 | 1.000 | 0.317 |
| *GRIN1* | -3356.58 | -3356.69 | 0.219 | 0.640 | -3356.69 | -3356.65 | 0.068 | 0.794 |
| *GRIN2A* | -6315.34 | -6315.40 | 0.133 | 0.715 | -6315.40 | -6315.38 | 0.044 | 0.834 |
| *GRIN2B* | -6258.06 | -6258.43 | 0.750 | 0.387 | -6258.43 | -6257.80 | 1.264 | 0.261 |
| *GRIN2C* | -4691.07 | -4691.73 | 1.329 | 0.249 | -4691.73 | -4691.64 | 0.177 | 0.674 |
| *GRIN2D* | -3042.00 | -3042.39 | 0.775 | 0.379 | -3042.39 | -3042.25 | 0.288 | 0.591 |
| *GRIN3A* | -5077.78 | -5078.47 | 1.372 | 0.241 | -5078.47 | -5078.23 | 0.475 | 0.491 |
| *GRIN3B* | -925.37 | -925.40 | 0.056 | 0.813 | -925.40 | -925.40 | 0.000 | 0.986 |
| *GRM1* | -5275.96 | -5276.10 | 0.274 | 0.600 | -5276.10 | -5276.09 | 0.014 | 0.906 |
| *GRM2* | -3775.37 | -3775.40 | 0.059 | 0.808 | -3775.40 | -3775.25 | 0.301 | 0.584 |
| *GRM3* | -3809.91 | -3810.97 | 2.129 | 0.145 | -3810.97 | -3809.36 | 3.223 | 0.073 |
| *GRM4* | -3746.72 | -3746.90 | 0.354 | 0.552 | -3746.90 | -3745.66 | 2.474 | 0.116 |
| *GRM5* | -5162.53 | -5162.89 | 0.713 | 0.398 | -5162.89 | -5162.24 | 1.298 | 0.255 |
| *GRM6* | -3211.91 | -3213.16 | 2.505 | 0.113 | -3213.16 | -3212.44 | 1.434 | 0.231 |
| *GRM7* | -3781.81 | -3792.00 | 20.371 | 0.000 * | -3781.95 | -3780.15 | 3.604 | 0.058 |
| *GRM8* | -3948.44 | -3947.63 | 1.615 | 0.204 | -3947.63 | -3946.81 | 1.631 | 0.202 |

The *p* value cut-off after correcting for multiple tests according to Bonferroni is 0.001. * indicates statistical significance.
