# Supplementary material for: The identification and functional implications of human-specific "fixed" amino acid substitutions in the glutamate receptor family
Source: BMC Evol Biol. 2009 Sep 8;9:224. doi: 10.1186/1471-2148-9-224 (PMC2753569; doi:10.1186/1471-2148-9-224)
Supplement: Additional file 2 — Results for the improved branch site model. The table presents likelihood ratios and p value for the improved branch site test. [file 1471-2148-9-224-S2.doc]

**Additional file 2 - Results for the improved branch site model**

| Type | | Gene | Length (bp) | Human | | | |  | Chimpanzee | | | |
| --- | --- | --- | --- | --- | --- | --- | --- | --- | --- | --- | --- | --- |
|  |  |  |  | M2 | Ma | − 2log(Λ) | *p* value* (*df* = 1) |  | M2 | Ma | − 2log(Λ) | *p* value * (*df* = 1) |
| Ionotropic | NMDA | *GRIN1* | 2430 | -3354.94 | -3354.94 | 0.00001 | 0.998 |  | -3354.94 | -3354.94 | 0.00002 | 0.996 |
|  |  | *GRIN2A* | 4299 | -6315.34 | -6315.34 | 0.00000 | 0.999 |  | -6315.40 | -6315.38 | 0.04236 | 0.837 |
|  |  | *GRIN2B* | 4452 | -6258.43 | -6258.43 | 0.00039 | 0.984 |  | -6257.80 | -6257.80 | 0.00043 | 0.983 |
|  |  | *GRIN2C* | 3252 | -4691.07 | -4691.07 | 0.00095 | 0.975 |  | -4691.65 | -4691.65 | 0.00000 | 0.999 |
|  |  | *GRIN2D* | 2241 | -3042.39 | -3042.39 | 0.00000 | 0.998 |  | -3042.39 | -3042.39 | 0.00000 | 0.998 |
|  |  | *GRIN3A* | 3345 | -5076.28 | -5076.28 | 0.00000 | 1.000 |  | -5077.23 | -5077.22 | 0.01239 | 0.911 |
|  |  | *GRIN3B* | 651 | -925.38 | -925.38 | 0.00000 | 1.000 |  | -925.40 | -925.40 | 0.00000 | 1.000 |
|  | AMPA | *GRIA1* | 2718 | -3904.80 | -3904.80 | 0.00032 | 0.986 |  | -3904.80 | -3904.80 | 0.00024 | 0.988 |
|  |  | *GRIA2* | 2649 | -3763.93 | -3763.93 | 0.00001 | 0.997 |  | -3763.93 | -3763.93 | 0.00001 | 0.997 |
|  |  | *GRIA3* | 2682 | -3765.00 | -3765.00 | 0.00344 | 0.953 |  | -3762.53 | -3762.15 | 0.76418 | 0.382 |
|  |  | *GRIA4* | 2706 | -3794.65 | -3793.43 | 2.44177 | 0.118 |  | -3794.97 | -3794.97 | 0.00016 | 0.990 |
|  | Kainate | *GRIK1* | 2757 | -4010.75 | -4010.75 | 0.00002 | 0.996 |  | -4010.75 | -4010.75 | 0.00070 | 0.979 |
|  |  | *GRIK2* | 2445 | -3456.57 | -3456.57 | 0.00024 | 0.988 |  | -3456.57 | -3456.57 | 0.00001 | 0.997 |
|  |  | *GRIK3* | 2757 | -3946.40 | -3946.40 | 0.00000 | 1.000 |  | -3946.70 | -3946.70 | 0.00000 | 1.000 |
|  |  | *GRIK4* | 2868 | -3973.17 | -3973.17 | 0.00000 | 0.998 |  | -3973.50 | -3973.50 | 0.00000 | 1.000 |
|  |  | *GRIK5* | 2937 | -4100.49 | -4100.49 | 0.00001 | 0.998 |  | -4103.57 | -4103.57 | 0.00110 | 0.974 |
|  | Delta | *GRID1* | 3027 | -4310.27 | -4310.27 | 0.01910 | 0.890 |  | -4313.07 | -4313.07 | 0.00000 | 0.999 |
|  |  | *GRID2* | 3021 | -4401.45 | -4401.45 | 0.00005 | 0.995 |  | -4400.69 | -4400.69 | 0.00624 | 0.937 |
| Metabotropic | | *GRM1* | 3582 | -5276.10 | -5276.10 | 0.00000 | 1.000 |  | -5276.10 | -5276.10 | 0.00001 | 0.997 |
|  |  | *GRM2* | 2616 | -3775.37 | -3774.85 | 1.02690 | 0.311 |  | -3775.25 | -3775.25 | 0.00171 | 0.967 |
|  |  | *GRM3* | 2637 | -3809.91 | -3809.89 | 0.03355 | 0.855 |  | -3809.37 | -3809.37 | 0.00003 | 0.995 |
|  |  | *GRM4* | 2664 | -3746.90 | -3746.90 | 0.00000 | 1.000 |  | -3745.67 | -3745.67 | 0.00000 | 0.999 |
|  |  | *GRM5* | 3507 | -5162.89 | -5162.89 | 0.00004 | 0.995 |  | -5162.89 | -5162.89 | 0.00001 | 0.997 |
|  |  | *GRM6* | 2160 | -3210.47 | -3210.47 | 0.00000 | 1.000 |  | -3211.63 | -3211.52 | 0.20858 | 0.648 |
|  |  | *GRM7* | 2586 | -3781.82 | -3781.82 | 0.00000 | 1.000 |  | -3780.17 | -3780.17 | 0.00008 | 0.993 |
|  |  | *GRM8* | 2724 | -3947.44 | -3947.44 | 0.00000 | 0.999 |  | -3946.81 | -3946.81 | 0.00124 | 0.972 |

**p* values for Test 2 in Zhang, J., Nielsen, R. and Yang, Z. (2005) [18].
