# Supplementary material for: The identification and functional implications of human-specific "fixed" amino acid substitutions in the glutamate receptor family
Source: BMC Evol Biol. 2009 Sep 8;9:224. doi: 10.1186/1471-2148-9-224 (PMC2753569; doi:10.1186/1471-2148-9-224)
Supplement: Additional file 3 — The source of genomic GluR sequences for humans, chimpanzees, and macaques. The table shows the data source of genomic GluR sequences. [file 1471-2148-9-224-S3.doc]

**Additional file 3 - The source of genomic GluR sequences for humans, chimpanzees, and macaques**

| Type | | Gene | Human | Chimpanzee  (GenBank accession number) | Macaque |
| --- | --- | --- | --- | --- | --- |
| Ionotropic | NMDA | *GRIN1* | ENST00000315048 | This study (AB514214) | UCSC |
|  |  | *GRIN2A* | ENST00000330684 | This study (AB514215) | UCSC |
|  |  | *GRIN2B* | ENST00000279593 | UCSC | UCSC |
|  |  | *GRIN2C* | ENST00000293190 | This study (AB514216) | UCSC |
|  |  | *GRIN2D* | ENST00000263269 | This study (AB514217) | UCSC |
|  |  | *GRIN3A* | ENST00000361820 | This study (AB514218) | UCSC |
|  |  | *GRIN3B* | ENST00000234389 | This study (AB514219) | UCSC |
|  | AMPA | *GRIA1* | ENST00000285900 | UCSC | UCSC |
|  |  | *GRIA2* | ENST00000264426 | UCSC | UCSC |
|  |  | *GRIA3* | ENST00000264357 | This study (AB514205) | UCSC |
|  |  | *GRIA4* | ENST00000282499 | This study (AB514206) | UCSC |
|  | Kainate | *GRIK1* | ENST00000309434 | This study (AB514207) | UCSC |
|  |  | *GRIK2* | ENST00000296893 | This study (AB514208) | UCSC |
|  |  | *GRIK3* | ENST00000296212 | This study (AB514209) | UCSC |
|  |  | *GRIK4* | ENST00000278723 | This study (AB514210) | UCSC |
|  |  | *GRIK5* | ENST00000262895 | This study (AB514211) | UCSC |
|  | Delta | *GRID1* | ENST00000327946 | This study (AB514212) | UCSC |
|  |  | *GRID2* | ENST00000282020 | This study (AB514213) | UCSC |
| Metabotropic | | *GRM1* | ENST00000282753 | This study (AB514220) | UCSC |
|  |  | *GRM2* | ENST00000296479 | This study (AB514221) | UCSC |
|  |  | *GRM3* | ENST00000361669 | UCSC | UCSC |
|  |  | *GRM4* | ENST00000266007 | This study (AB514222) | UCSC |
|  |  | *GRM5* | ENST00000305447 | UCSC | UCSC |
|  |  | *GRM6* | ENST00000231188 | This study (AB514223) | UCSC |
|  |  | *GRM7* | ENST00000357716 | This study (AB514224) | UCSC |
|  |  | *GRM8* | ENST00000341617 | This study (AB514225) | UCSC |
