# Supplementary material for: The identification and functional implications of human-specific "fixed" amino acid substitutions in the glutamate receptor family
Source: BMC Evol Biol. 2009 Sep 8;9:224. doi: 10.1186/1471-2148-9-224 (PMC2753569; doi:10.1186/1471-2148-9-224)
Supplement: Additional file 4 — Human DNA samples used in the polymorphism survey. The table presents Coriell numbers of human DNA samples for genotyping in human populations. [file 1471-2148-9-224-S4.doc]

**Additional File 4 - Human DNA samples used in the polymorphism survey**

| ID# | Population | Population | Catalog ID | Sex |
| --- | --- | --- | --- | --- |
| 1 | Asian | Japanese | J01 | M |
| 2 |  |  | J02 | M |
| 3 |  |  | J03 | M |
| 4 |  |  | J04 | M |
| 5 |  |  | N01 | F |
| 6 |  |  | N02 | F |
| 7 |  |  | N03 | F |
| 8 |  |  | N04 | F |
| 9 |  | Thai | TC001 | M |
| 10 |  |  | TC002 | M |
| 11 |  |  | TC003 | M |
| 12 |  |  | TC004 | M |
| 13 |  |  | TC151 | F |
| 14 |  |  | TC152 | F |
| 15 |  |  | TC153 | F |
| 16 | Europe | Italian | NA17321 | F |
| 17 |  |  | NA17322 | F |
| 18 |  |  | NA17323 | F |
| 19 |  |  | NA17324 | M |
| 20 |  |  | NA17325 | M |
| 21 |  |  | NA17326 | M |
| 22 |  |  | NA17327 | F |
| 23 |  |  | NA17328 | F |
| 24 |  |  | NA17329 | M |
| 25 |  |  | NA17330 | F |
| 26 |  | Northern European | NA17001 | F |
| 27 |  |  | NA17002 | F |
| 28 |  |  | NA17003 | M |
| 29 |  |  | NA17004 | F |
| 30 |  |  | NA17005 | F |
| 31 |  |  | NA17006 | F |
| 32 |  |  | NA17007 | M |
| 33 |  |  | NA17008 | M |
| 34 |  |  | NA17009 | F |
| 35 |  |  | NA17010 | M |
| 36 | Africa | North of Sahara | NA17378 | M |
| 37 |  |  | NA17379 | F |
| 38 |  |  | NA17380 | F |
| 39 |  |  | NA17381 | M |
| 40 |  |  | NA17382 | M |
| 41 |  |  | NA17383 | M |
| 42 |  |  | NA17384 | M |
| 43 |  | South of Sahara | NA17341 | F |
| 44 |  |  | NA17342 | M |
| 45 |  |  | NA17343 | M |
| 46 |  |  | NA17344 | F |
| 47 |  |  | NA17345 | M |
| 48 |  |  | NA17346 | M |
| 49 |  |  | NA17347 | M |
| 50 |  |  | NA17348 | F |
| 51 |  |  | NA17349 | M |
| 52 |  | African American | NA17031 | F |
| 53 |  |  | NA17032 | F |
| 54 |  |  | NA17033 | F |
| 55 |  |  | NA17034A | F |
| 56 |  |  | NA17035A | F |
| 57 |  |  | NA17036 | F |
| 58 |  |  | NA17037 | F |
| 59 |  |  | NA17038 | F |
| 60 |  |  | NA17039 | F |
| 61 |  |  | NA17040A | F |
| 62 | Middle East | Middle Eastern | NA17041*A1 | F |
| 63 |  |  | NA17042 | F |
| 64 |  |  | NA17043 | M |
| 65 |  |  | NA17044 | F |
| 66 |  |  | NA17045 | M |
| 67 |  |  | NA17046*A1 | M |
| 68 |  |  | NA17047*A1 | M |
| 69 |  |  | NA17048 | F |
| 70 |  |  | NA17049*A1 | M |
| 71 |  |  | NA17050*A1 | M |
| 72 | South America | South America | NA17311 | F |
| 73 |  |  | NA17312 | F |
| 74 |  |  | NA17313 | M |
| 75 |  |  | NA17314 | M |
| 76 |  |  | NA17315 | M |
| 77 |  |  | NA17316 | M |
| 78 |  |  | NA17318 | M |
| 79 |  |  | NA17319 | F |
| 80 |  |  | NA17320 | F |
