# Supplementary material for: The identification and functional implications of human-specific "fixed" amino acid substitutions in the glutamate receptor family
Source: BMC Evol Biol. 2009 Sep 8;9:224. doi: 10.1186/1471-2148-9-224 (PMC2753569; doi:10.1186/1471-2148-9-224)
Supplement: Additional file 5 — Primers and PCR conditions. The table shows the primer sequences and PCR conditions for genotyping in primates. [file 1471-2148-9-224-S5.doc]

**Additional file 5 - Primers and PCR conditions**

| ID# | Gene | Amino acid substitution | Primer A | Primer B | Size | PCR condition |
| --- | --- | --- | --- | --- | --- | --- |
| 1 | *GRIA3* | P590L | CCCCTGGCTTATGAAATCTG | CATAAAGGCACCCAAGGAAA | 204 |  |
| 2 | *GRIA4* | S5C | GCCATCGTCTTCAATGCTT | GAGTCCCCAAAATCCAGAAA | 143 |  |
| 3 | *GRIK3* | S310A | GATCTCTACGCTTTAGACCTGGA | CATCATCACTCCATCCAGCA | 174 |  |
| 4 | *GRIK3* | V419I | CTGTCTGTGTCCATCCAGGT | CAGCACTGTGGTGACAATGA | 132 |  |
| 5 | *GRIK4* | H403R | GAGGAGGACCATCGAGCTAA | CAATGATGAGGCCACAGATGA | 140 | 2.5mM 60Cx35 |
| 6 | *GRIK5* | L298P | TCCTGCATCTGGACGGTAT | TGACTGGGTATCTGTGTGCTC | 175 |  |
| 7 | *GRIK5* | I809V | GAGGAGGACCATCGAGCTAA | CAATGATGAGGCCACAGATGA | 183 | 2.5mM 58.1Cx35 |
| 8 | *GRIK5* | A922T | AGCAACGGCAAGCTCTACTC | GGAACCAAAGGCAAAATCG | 294 |  |
| 9 | *GRIK5* | V956A |
| 10 | *GRID1* | T295M | TAGTGCCCTTGGAAGGATGAC | CTGCAGCATCTGGAGGTAGC | 136 | 2.5mM 60Cx35 |
| 11 | *GRID1* | M628V | GGCGAATCTTCCGTGAACTC | GTCCATCCTGGACACTGTGA | 126 | 2.5mM 60Cx35 |
| 12 | *GRID2* | S11F | CCAAGAGAATCGGCATAGGA | ACGAGCTGCACCAACACTTT | 137 |  |
| 13 | *GRM2* | A6G | TTGCCTTCGCTGCTTCTAAT | ACAGGACCACAGTCCTCTGCTG | 230 |  |
| 14 | *GRM2* | A248V | CTCCGCTTCTTCAACTGGAC | AGAACGGGTGAACAGGACAG | 222 |  |
| 15 | *GRM3* | M547V | GAATATGCAACCAGGGGATGT | GGCGTCTTCCCACCTGATGT | 168 |  |
| 16 | *GRM3* | S551P | GGATGTCTGCTGCTGGATTTG | AGGGTGTGTTGTTGTGCTTGA | 243 |  |
| 17 | *GRM3* | M593T |
| 18 | *GRM4* | L19F | AGAGAGGCTTGGGCTGGTG | GTGGATGCCCTTTTCCTTCT | 221 |  |
| 19 | *GRM6* | Q59P | CTGAGCTAACTCCCCAGAGC | GCTCCAGCGCGTAGGTGT | 208 |  |
| 20 | *GRM6* | P141T | GCTGCTGGACACCTGCTC | CATGATGGAGACGGAGCTGG | 195 | FailSafe-G |
| 21 | *GRM6* | D380E | GAACAACCGCAGGAACATCTG | GCAGCTCTCACCTGTGCATT | 210 |  |
| 22 | *GRM6* | M442T | GTTTGTGATTGATGCGGTGTA | GCGGACAGCTCGAATGTACT | 142 | 2.5mM 60Cx35 |
| 23 | *GRM6* | Y612H | CTGTCCTGGGGACATGAG | GAGCAGGGCAGAGTAGCTGA | 335 | 2.5mM 60Cx35 |
| 24 | *GRM6* | A650G |
| 25 | *GRM6* | M714V | TGATGCAGTGTTGGCTCCT | GCAGCCGATGAGAGACAGAT | 161 | 2.5mM 60Cx35 |
| 26 | *GRM6* | V839I | CATCCAGACAACCACGCTAA | CACCTGCCCTGCTACTTGTG | 204 | 2.5mM 60Cx35 |
| 27 | *GRM6* | A877D |
| 28 | *GRM7* | A520P | GGGGTAAAGGAGTCCGAGA | GCAATGCTGGCATGTCATCT | 157 |  |
| 29 | *GRM8* | R268C | ACGTGAACCAAGACCTGGAG | AAGGCAGTCTGTTATTGGAAGG | 163 |  |
| 30 | *GRM8* | G327V | GGAGGATATTGGAAGCAGCA | TTTGCCTTTGAGTTCAGATCC | 215 | 2.5mM 58C x35 |
| 31 | *GRM8* | V653I | GCTTCAGGACGCGAACTTAG | CACCAGCTGAGATGCTGGAC | 245 |  |
| 32 | *GRIN2A* | S906N | GCATTCATGGAGTGCACAT | GCAGCTCTTTTGGGTGAGT | 157 |  |
| 33 | *GRIN2A* | A1006V | CGGCAGAAGGATAACCTCAA | CAGTCCTTGGGGTATTTGG | 386 |  |
| 34 | *GRIN2A* | H1080P |
| 35 | *GRIN2A* | F1158L | CCCTACCAGGATCCCAGTGA | GGTGGCTGGGTTACCTGTCT | 338 |  |
| 36 | *GRIN2A* | H1173Q |
| 37 | *GRIN2A* | M1221L |
| 38 | *GRIN2B* | N1294T | TACCCTCAGAGCCCGACTAA | GCCAGCTGACATCTCAAACA | 195 |  |
| 39 | *GRIN2C* | P23M | CTGTTGCTCACCTCGCTCTT | GGTCTGGGAGGAGATGAAGTC | 321 | 2.5mM 58C x35 |
| 40 | *GRIN2C* | T71N |
| 41 | *GRIN2C* | H89R |
| 42 | *GRIN2C* | D100G | CAGAGCTTCCTGGACCTACC | CACATGGGTCTGGGAGGAGA | 195 | 1.25mM, 62Cx35 |
| 43 | *GRIN2C* | A596S | CCTCTTGCCCCCAGAGTC | GTAGCTGGCGAGGAAGATGA | 172 |  |
| 44 | *GRIN2C* | S851T | GGGATCTGCCAGAATGAGAA | GCTGAAAGCCAGCAGGAAGT | 195 |  |
| 45 | *GRIN2C* | Q898R | GGCATCTACAGCTGCTTCAG | CGGTCCAGGGAGCTGCTTA | 161 |  |
| 46 | *GRIN2C* | S933P | CACTCGCACCATCGAGAAT | GGGAGACGTCGGACAGG | 230 | FailSafe-G |
| 47 | *GRIN2C* | G1144S | GATGTGCTTGCCGATCTACC | CAGTCCCCCACTGTCTCTGT | 247 | FailSafe-A |
| 48 | *GRIN2C* | R1221C |
| 49 | *GRIN3A* | S30G | AGCAGGGTCTGTCTGCTGTT | TGGTCCAGGGCTGCAAGT | 147 | 2.5mM 58.1Cx35 |
| 50 | *GRIN3A* | D71G | AGCAGGGTCTGTCTGCTGTT | GCTCCGGCTCATCCCTCT | 217 | FailSafe-G |
| 51 | *GRIN3A* | P93L | CTTGCAGCCCTGGACCAC | TGGCCATCACTACTTCCAAAG | 299 | FailSafe-G |
| 52 | *GRIN3A* | A119T |
| 53 | *GRIN3A* | A121T |
| 54 | *GRIN3A* | V138M |
| 55 | *GRIN3A* | E340K | TCCAAGTTCCACCTTGGTTC | GTTCCTCCACATTCTGGGAAT | 235 |  |
| 56 | *GRIN3A* | A885S | TCTTCTAGGATACGGCATTGG | AGCAAAACTTCTCTTGCCAC | 171 |  |
| 57 | *GRIN3A* | I988V | TGGTTCTTTGTTCCCTGACC | TTGCCCTGATTTGATCTGAA | 234 | 2.5mM 54Cx35 |
| 58 | *GRIN3A* | R1059L | GACAACCGACGGAAATACAT | ACGGATCACCTGAATCTGCT | 207 |  |
| 59 | *GRIN3B* | P17S | CAACTTTGCGATGGAGTTTG | ACCAGCTCCAAGCTCAGGT | 237 | FailSafe-G |
| 60 | *GRIN3B* | G175S | GCTGCTGGATGTGCTGGT | GATGTCACAGCCGAGGAGGA | 323 |  |
| 61 | *GRIN3B* | E229G |
| 62 | *GRIN3B* | A272V | ACTGGCTGTTGGGGACACC | GCAGGTCCCCGCAGTTGA | 522 | FailSafe-G |
| 63 | *GRIN3B* | I296T |
| 64 | *GRIN3B* | W414R | TTTAAGGTGTGGAGCCTTCG | GTTCCAACAGCGTTACCACAC | 196 |  |
| 65 | *GRIN3B* | A468V | CACCCTGGACGCACTGTT | GGCGGAGTTGATACTGAAGC | 250 | FailSafe-L |
| 66 | *GRIN3B* | R473C |
| 67 | *GRIN3B* | L499I |
| 68 | *GRIN3B* | T577M | CTCACAGGTGGTGGACTTCAC | GAAGAGGATGGCGTAGCACA | 271 |  |
| 69 | *GRIN3B* | Y595C |
| 70 | *GRIN3B* | R598C |
| 71 | *GRIN3B* | V613I |
| 72 | *GRIN3B* | R727H | CCGAGGCGTACATCAAGAAG | ATGAAGGCGTTGAGCTTGG | 206 | PrimeStar |
| 73 | *GRM1* | S993P | GTAGCCCTTCCATGGTGGT | CTGGTCCATCAGCGATTTCT | 185 |  |
| 74 | *GRM1* | L1089P | GGATTTTCACGCGGTGCT | GCTTAAACCTCTCGCTGTCGT | 176 | 2.5mM 60Cx35 |
| 75 | *GRM6* | GD125-126del | GCGGGCAGCTGAAGAAGGA | GGCGACCATGATGGAGAC | 196 | FailSafe-G |
| 76 | *GRIN2C* | RALPER1021-1026del | GTCCGACGTCTCCCGAGT | GTCGGCTCGAGGAAAGGAGC | 145 | FailSafe-L |
| 77 | *GRIN2C* | PPE1055-1057del | GCGCTGTCACTACAGCTCCT | CAGACGTGCTGTCTGTGCT | 375 | FailSafe-G |
| 78 | *GRIN2C* | AH1164-1165del | GATGTGCTTGCCGATCTACCG | GGCTACCCTGCTGATCTCGT | 247 |  |

FailSafe: FailSafe™ PCR System (EPICENTRE Biotechnologies, Madison, WI) is the kit including various types of PCR buffer. Letter indicates a specific buffer in the kit.
